# Knotting together of gastric and tracheal tubes inserted under general anesthesia: A CARE-compliant case report

**DOI:** 10.1097/MD.0000000000031203

**Published:** 2022-10-28

**Authors:** Jingjing Deng, Ting Pan, Hui Li, Qing Lin, Yuenong Zhang

**Affiliations:** a Department of Surgery and Anesthesia, The Third Affiliated Hospital of Sun Yatsen University Yuedong Hospital, Meizhou city, Guangdong Province, China.

**Keywords:** general anesthesia, knotting, tracheal tubes

## Abstract

Gastric tube implantation is a routine part of preoperative preparation. Indwelling gastric tubes in patients under general anesthesia maintain gastrointestinal decompression and prevent gastrointestinal expansion.

**Patient concerns:**

Gastric tube insertion can be associated with many complications, of which gastric tube knotting is a rare and often overlooked complication.

**Diagnoses:**

Knotting together of gastric and tracheal tubes.

**Interventions:**

During the operation, the gastric tube was explored by endoscope and hand.

**Lessons:**

Rare complications of knotted gastric and endotracheal tubes are identified and treated promptly.

**Conclusion:**

We recommend that the gastric tube be intubated first before insertion of the endotracheal tube, and visualization tools should be used in time if the insertion of the gastric tube is unsuccessful.

## 1. Introduction

Gastric tube implantation is part of the routine preparation for surgical procedures. In patients under general anesthesia, an indwelling gastric tube enables gastrointestinal decompression and prevents gastrointestinal expansion.^[1]^ In addition, the color, quality, and quantity of the drainage fluid from the gastric tube are helpful for diagnosis and treatment.^[2]^ Gastric tubes are often inserted via the nose in a blind manner. Indwelling gastric tubes can lead to nasal complications (including ulcer and nasal necrosis), esophageal complications (including erosion and perforation), pulmonary complications (including aspiration, and bronchial and lung injury), and tube-related complications (including tube rupture and knotting).^[1,3]^ Gastric tube knotting is a rare and often overlooked complication. If undiagnosed, gastric tube knotting may lead to serious damage on tube removal.^[1]^

Here, we report an extremely rare case of knotting of a gastric tube with a tracheal tube in a patient under general anesthesia.

## 2. Case report

This case report was approved by the Ethics Committee of the Third Affiliated Hospital of Sun Yat-sen University-Yuedong Hospital. A 67-year-old woman (height, 140 cm; weight, 40 kg) was admitted to our hospital with a 1-week history of yellowish skin and eyes. She had no special medical history. She was diagnosed with a malignant bile duct tumor, and was scheduled to undergo cholecystectomy, extrahepatic bile duct resection, cholangiojejunostomy, and hilar lymph node dissection under general anesthesia. After the induction of anesthesia, we first inserted a tracheal tube (internal diameter, 7.0 mm; Henan Camel Medical Equipment Group Co. Ltd, Changyuan County, Xinxiang City, Henan Province, China) and then inserted a silicone gastric tube (F16; diameter, 5.3 mm; Rugao Hengkang Medical Equipment Co. Ltd.Rugao City, Nantong City, Jiangsu Province, China). The gastric tube with a catheter core was blindly inserted through the right nostril. There was slight resistance after insertion up to 10 cm. However, after changing the patient’s body position and bending her head slightly, the resistance disappeared. After insertion up to 70 cm, the catheter core was successfully removed, and the gastric tube was slightly withdrawn to 55 cm.

**Figure 1.**
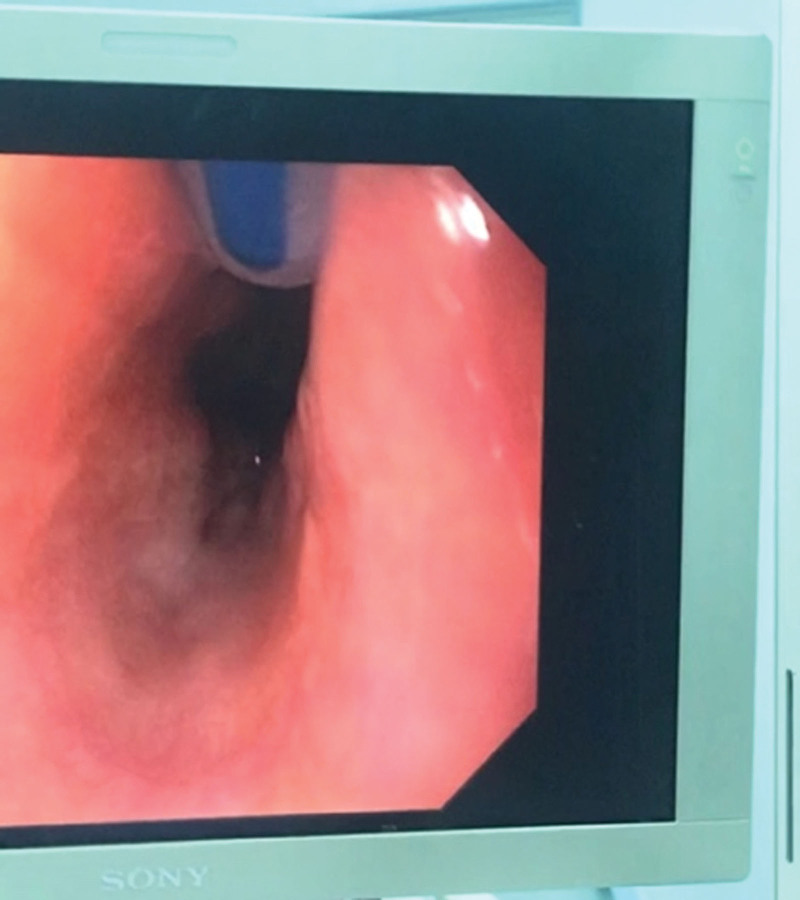
The tip of the gastric tube had entered the upper esophagus.

**Figure 2.**
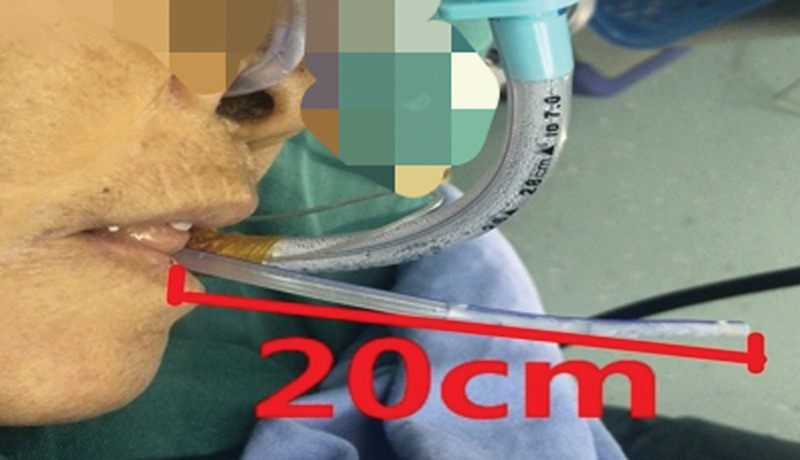
The gastric tube is pulled out of the esophagus, up to a length of 20 cm, via the mouth.

**Figure 3.**
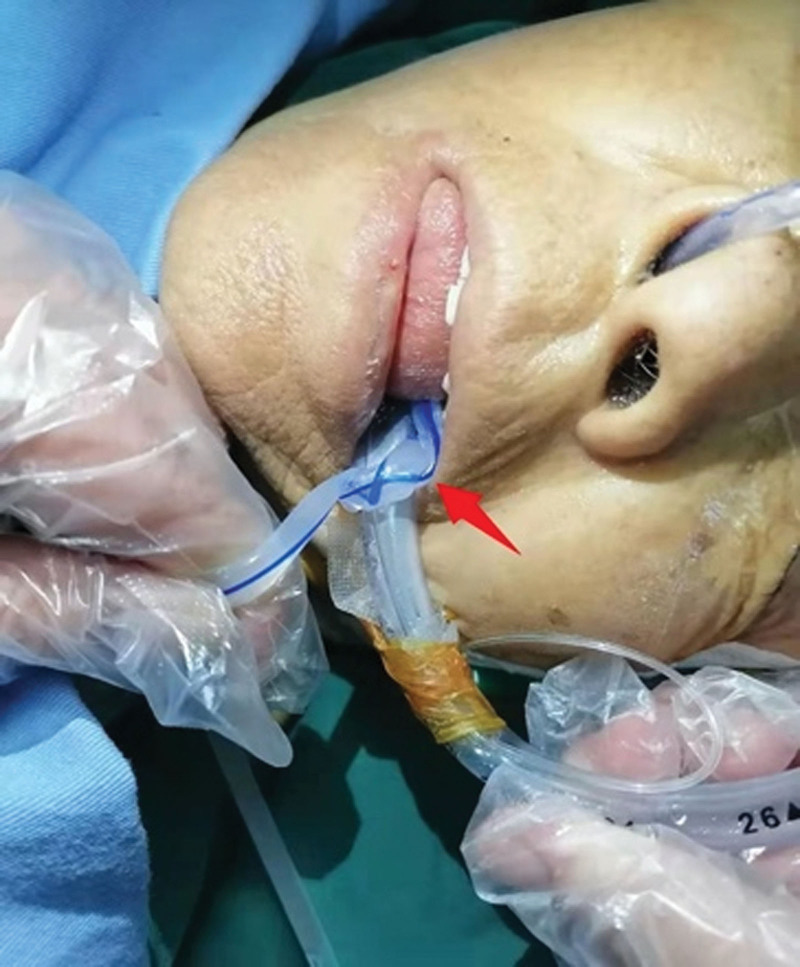
Knotting of the gastric and tracheal tubes (arrow).

After connecting the tube with a negative pressure bottle, there was no gastric fluid drainage. During the surgery, the patient was diagnosed with hilar cholangiocarcinoma, and resection of the hilar cholangiocarcinoma was performed. The operation was successful. The intraoperative blood loss was approximately 150 mL. Before closing the abdomen, we were unable to detect the gastric tube in the stomach, so the depth of the tube was adjusted. When the gastric tube was withdrawn to 40 cm, it could not be pulled out or reinserted. Nasal fiberoptic bronchoscopy was performed, which confirmed that the gastric tube had entered the esophagus. Next, oral gastroscopy was performed, which showed that the front end of the gastric tube was located in the upper part of the esophagus (Fig. 1). Then, the position of the gastric tube was manually checked by inserting fingers into the oral cavity. The front end of the gastric tube was successfully pulled out through the oral cavity, up to a length of approximately 20 cm (Fig. 2). However, pulling of the front end of the gastric tube also resulted in the removal of the tracheal tube. We then discovered that the gastric tube and the tracheal tube were knotted together; the knot was located at the 18-cm mark of the tracheal tube (Fig. 3).

## 3. Discussion

The potential factors contributing to gastric tube knotting are unclear. The swallowing reflex disappears under general anesthesia. In anesthetized patients, when the gastric tube is inserted in the supine position, the tube is usually prevented from inadvertently entering the trachea by a previously inserted tracheal tube. However, posterior tilting of the catheter and cuff of the tracheal tube may interfere with the advance of the gastric tube.^[4]^

In the present case, when the gastric tube was blindly inserted through the nose, resistance was encountered after 10 cm of insertion, and the gastric tube could not be successfully inserted into the esophagus. We adjusted the patient’s position, and were able to continue with the insertion. We can now surmise that the gastric tube must have circled the tracheal tube in the oral cavity, and then entered the esophagus. This phenomenon may be related to the patient’s small stature and small oral cavity space. When we attempted to pull out the gastric tube, the loop must have tightened, resulting in the knotting together of the gastric and tracheal tubes.

Although we encountered resistance while inserting the gastric tube, we did not adjust or pull out the tube for re-implantation. Instead, we continued with the implantation. After 70 cm of insertion, the catheter core of the gastric tube was removed, and the tube was slightly withdrawn to 55 cm. After connecting the negative pressure bottle, there was no gastric fluid drainage. Moreover, during the entire surgery, the gastric tube was not actively checked, and the possibility of the knotting together of the gastric and tracheal tubes was not considered.

This case highlights the importance of checking whether the gastric tube is successfully indwelling. Bedside confirmatory tests for correct gastric tube placement include the following: observing whether gastric fluid is withdrawn through the gastric tube, placing the external port of the gastric tube in water to observe bubbles escaping, listening to the sound of breaths passing through water, checking the amount of gastric fluid drainage, measuring the residual gastric volume, and testing the Pondus Hydrogenii of the gastric aspirate.^[5]^ Other confirmatory tests include X-ray or fluoroscopy examination, colorimetry, and measuring the inserted length of the gastric tube.^[6]^ Studies have shown that video laryngoscope-assisted gastric tube placement can improve the success rate of insertion and reduce operation time.^[7]^

In the present case, the gastric tube could not be detected in the stomach before the closure of the abdomen. We tried to move the gastric tube, and found that it could not be pulled out or reinserted after it had been withdrawn to 40 cm. When we attempted to pull out the gastric tube through the nose, we did not find that the tracheal tube moved together with it. This is different from gastric and tracheal tube knotting in the case of oral gastric tube insertion.^[8]^ Nasal fiberoptic bronchoscopy confirmed that the gastric tube had entered into the esophagus, and did not show knotting of the gastric and tracheal tubes. This is because the bronchoscope was not passed through the mouth, so it was impossible to show the condition of the gastric tube in the mouth. However, oral gastroscopy examination after the surgery also did not reveal the knot. This may be related to obstruction by the body of the tongue and the high position of the knot in the tracheal tube.

Cases of gastric tube knotting that went undetected during surgery have been reported before.^[3,5]^ Au-Truong X et al^[4]^ reported a case in which knotting of the gastric and tracheal tubes was found immediately after intubation. In their case, the tracheal tube and the knotted nasogastric tube were withdrawn together from the mouth; the gastric tube was then successfully inserted through the other nostil.^[4]^ When tracheal and gastric tubes are inserted together through the mouth^[8]^ or the nose,^[9]^ they may become knotted. In our case, we did not detect the knot during the operation. When a gastric tube cannot be pulled out during intubation, the possibility of tube knotting should be considered. Furthermore, laryngoscopy or video laryngoscopy can be used to check the condition of the oral cavity, and may reveal a knot.

In conclusion, after anesthesia induction, we suggest that gastric tube intubation be performed as the first step, prior to tracheal tube insertion. If there is resistance when inserting the gastric tube, the tube should be withdrawn and reinserted. A laryngoscope may be used to assist gastric tube intubation and may help avoid knotting of the gastric and tracheal tubes.

## Acknowledgments

We sincerely thank the Digestive Endoscopy Center of Yuedong Hospital, the Third Affiliated Hospital of Sun Yat-sen University for their help in the diagnosis and treatment of the patient.

## Author contributions

**Data curation:** Hui Li.

**Formal analysis:** Ting Pan.

**Project administration:** Yuenong Zhang.

**Resources:** Qing Lin.

**Writing – original draft:** Jingjing Deng.
